# Prevalence and therapeutic management of snakebite cases in the health facilities of the Bouenza department from 2009 to 2021, Republic of Congo

**DOI:** 10.11604/pamj.2022.42.139.35024

**Published:** 2022-06-21

**Authors:** Lise Bethy Mavoungou, Kate Jackson, Joseph Goma-Tchimbakala

**Affiliations:** 1 Département de Biologie, Institut National de Recherche en Sciences Exactes et Naturelles (IRSEN), Brazzaville, République du Congo,; 2 Asclepius Snakebite Foundation, 15223 E. Progress Place, Aurora, CO 80015, United States of America,; 3 Biology Department Whitman College, Walla Walla, WA 99362, United States of America,; 4 Ecole Nationale Supérieure d´Agronomie et de Foresterie, Université Marien NGOUABI (ENSAF, UMNG), Brazzaville, République du Congo

**Keywords:** Epidemiology, snakes, envenomation, treatment, Bouenza, Republic of Congo

## Abstract

**Introduction:**

few studies exist of snake bites in the Republic of Congo. This study reports epidemiological and management data on snake bites in the Bouenza department of the Republic of Congo.

**Methods:**

this is a retrospective and descriptive study based on questionnaire and analysis of files of snakebite victims over a period of 13 years (2009-2021). We collected data on incidence, age, gender, site of the bite, the season of the bite, deaths and treatment.

**Results:**

we identified 81 cases of bites recorded in 14 healthcare facilities: 54.32% of cases (44/81) at Nkayi Base Hospital; 11.11% (9/81) at Madingou Base Hospital; 1-5% (1-4/81) at each of the remaining facilities. Eight deaths were recorded in four health facilities. The sex ratio of snakebite victims was 1 (41 males: 40 females). The age most affected was 25-55 years (54.32% or 44/81). The lower limb was the bite site most reported at 13.98% (but in 84% or 68/81 cases the site was not recorded). More bites occurred during the rainy season (80.25%; 65/81). All victims received only symptomatic treatment based on antibiotics, anti-inflammatories and analgesics, as anti-venom serums are unavailable. The recorded incidence of snake bites in Bouenza was 18.62 per 100,000 of population.

**Conclusion:**

our study offers a preliminary report from a little-studied region. The incidence of snakebites recorded in Bouenza is lower than expected compared with studies from other African countries, and with earlier (20 years ago) studies from Congo. This may reflect incomplete record-keeping in under-resourced healthcare facilities.

## Introduction

Snakebites are a serious public health problem in tropical regions due to their high incidence and the severity of the clinical symptoms. They are not systematically reported in most countries [1]. The frequency of snakebites in Africa is largely unknown. However, it is generally accepted that the incidence of bites is 10 per 100,000 inhabitants in urban areas and can reach 400 bites per 100,000 inhabitants in rural areas [2]. Snakes only bite to defend themselves and protect their escape, and no species is aggressive in the sense that it attacks humans [3]. The bite is therefore the direct consequence of an encounter, accidental or intentional, between humans and snakes. In Congo Brazzaville, few studies have been devoted to snakebites. The existing data are too fragmentary to give a clear picture of the incidence of bites in the country. The first study on the estimation of morbidity due to snakebites dates back 35 years [4]. Studies continued with the observation of 3 bite victims for which the responsible snakes had been killed and preserved [5]. Recent studies are reported by the intensive care unit of the University Hospital Center of Brazzaville on two cases of viper envenomation [6]. The other studies were carried out in health facilities and the offices of village committees in six departments of the country, as well as in pharmacies and drug sales and purchase centres located in the city of Brazzaville [7,8]. During these studies, 165 cases of snake bites were counted, including 6 envenomations. Ten cases of snakebites were recorded in the department of Bouenza among the 165 cases of bites cited in the study [7]. Despite these studies, the department of Bouenza, like all the other departments of the Congo, remains poorly known from the point of view of the incidence and management of snakebites. This study aims to improve knowledge on the epidemiology and management of snakebites in healthcare facilities in Congo. This study focuses on the healthcare facilities of the department of Bouenza. To achieve this objective, surveys were carried out in the various healthcare facilities of 10 districts of the department of Bouenza.

## Methods

**Study design:** this is a retrospective and descriptive study carried out from August 11 to 30, 2021 to determine the prevalence of bite cases and having consisted of consulting the archives of healthcare facilities in the Department of Bouenza, in the Republic of Congo. The study focused on the analysis of hospital registers of triage and/or surgery services mentioning cases of snakebites received in these healthcare facilities over a period of thirteen years from 2009 to 2021. These are cases hospitalized or outpatient in public healthcare facilities. We also checked whether the victims had received treatment with antivenom or just palliative care.

**Figure 1:**
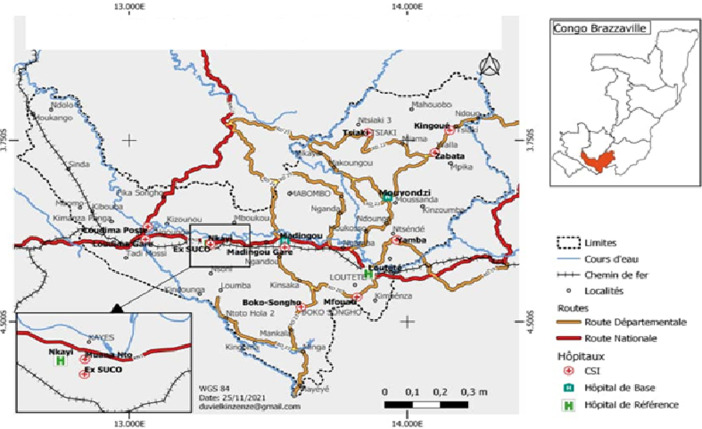
location of health facilities in Bouenza

**Study setting and population:** the study was conducted in healthcare facilities in the department of Bouenza. The department of Bouenza (4°5'40.85"S; 13°43'34.3"E) is located in the southern part of the country (Figure 1). It is one of the twelve departments of the Republic of Congo. It extends over 12,267 km^2^ (3.6% of the surface area of the Congo). It is bordered to the north by the department of Lékoumou, to the south by the Democratic Republic of Congo (DRC), to the east by the Department of Pool, and to the west by the Department of Niari. This zone is characterized by a transitional equatorial climate called Bas-Congolese or Sudano-Guinean [9]. The economic activity of the population is mainly agricultural. The two habitat types found in the Bouenza are forest and savannah. The Bouenza´s estimated total population was 434,925 in 2018 [10]. Participants in this study were all inpatients and outpatients in public health facilities who had been bitten by a snake.

**Variables:** records of all patients with snake bites from January 2009 to August 2021 were reviewed and data on age, gender, number of bites, parts of body bitten, antivenom use and mortality collected. Categorical variables were summarized in percentages and frequencies while continuous variables were represented as the mean and standard deviation.

### Data sources/ measurement

**Data collected:** we used information from hospital records from triage and/or surgery wards mentioning snakebite cases. We transcribed these data were on questionnaires. This retrospective survey based on 13-years of data was carried out using a questionnaire taking into account the characteristics of snakebite victims, the circumstances of the bite, the site of the bite, the management of the patient and of the type of envenomation, age, gender. Data were collected in the various healthcare facilities in 10 districts of the Bouenza department. For healthcare facilities that are difficult to access, such as the Integrated Health Centers (CSIs) in Mabombo, healthcare workers helped us fill out the questionnaires through telephone calls.

**Statistical analysis:** the data collected were entered into Microsoft Excel 2007 software, exported and analyzed using Statistical Package for Social Science (SPSS) version 22 and R version 3.6.1 (2019-07-05) software.

**Ethical considerations:** the global ethical rules relating to respect for confidentiality and the protection of patients´ personal data were taken into account during this work. The ethical clearance has been approved by the institutional ethics committee of the Congolese Foundation for Medical Research under No. 022/CIE/FCRM/2019.

**Funding sources:** this study was carried out with funding from the Ministry for Economic Cooperation and Development (BMZ) of the Federal Republic of Germany through the KfW (German Development Bank) on the basis of financial cooperation with the Coordination Organization for the fight against Endemics in Central Africa (OCEAC).

## Results

### Epidemiological aspects

**Sociodemographic data:** during the study period, we recorded 81 cases of snake bites in 15 health facilities. The bites are distributed as follows: 41 male cases and 40 female cases, i.e. a sex ratio of 1. The average age of the victims was 37.6 ±17 years, with a range from 4 to 70 years. Most bite cases occurred during the rainy season (80.25%). The profession of the patients was not specified in 89% of the cases. The overall fatality rate for snakebite was 9.9% (8/81) for all healthcare facilities. During the study period, the prevalence of bites was 0.81% (81/10000).

**Distribution by age:** we grouped ages into four categories as follows: children (4-14 years old), youth (15-24), adult (25-55) and the elderly (> 55 years old). Most bites (54.32 % or 44 cases) occurred in the adult (25-55) age group, followed by the elderly (55-70) with 18.52% (15 cases). Youths (15-25) made up (16.05% or 13 cases) and children of (4-15) represent 11.11% (or 9 cases).

**Figure 2:**
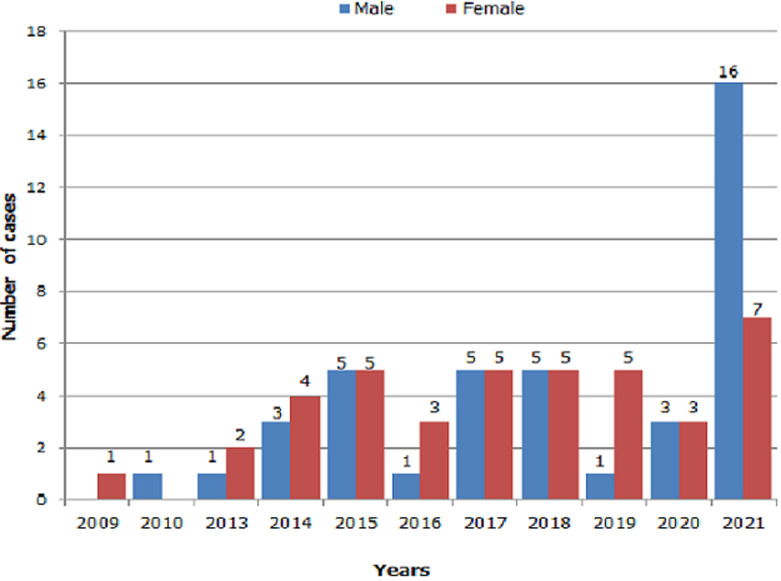
distribution according to gender and years

**Distribution by year and sex:** the breakdown of recorded bite cases by gender and year shows that in 2021 there were more men bitten than women. In 2015, 2017, 2018 and 2020, the number of victims according to gender is similar while in 2013, 2014, 2016, and 2019, more women were bitten than men. In 2009 no male victims were recorded and in 2010 no female victims were recorded (Figure 2).

**Figure 3:**
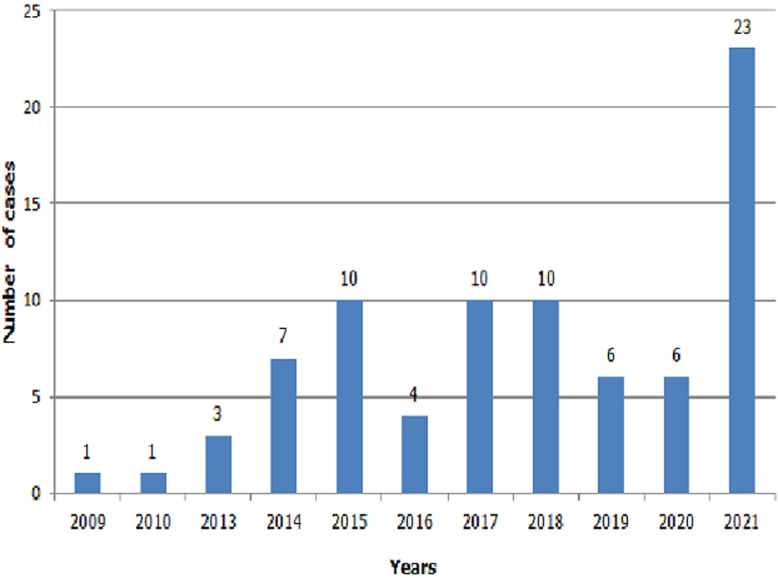
distribution according to years

**Distribution by year and incidence:** there were a total of 81 bites in all the study sites over the thirteen years study period. 23 cases occurred in 2021, 10 in 2018, 2017 and 2015. In other years, health facilities recorded fewer cases (Figure 3). During the study period, the incidence of snake bites in Bouenza was 18.62 per 100,000 population.

**Figure 4:**
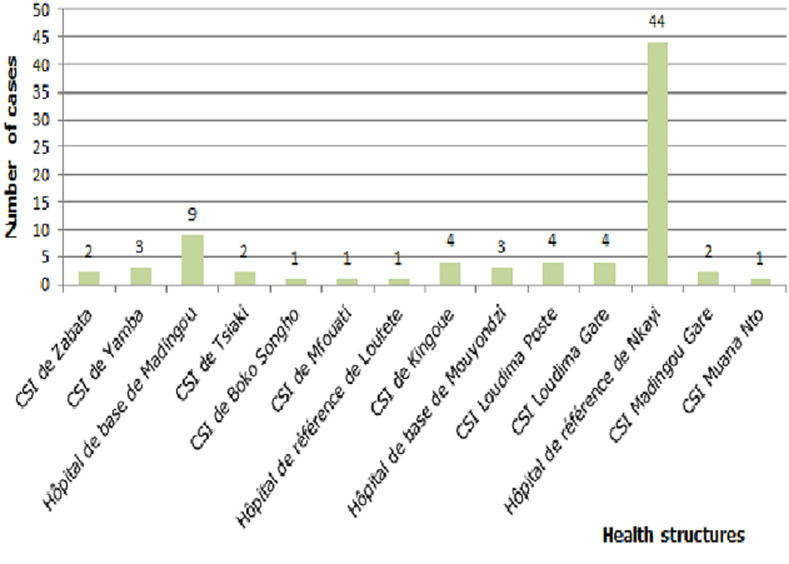
distribution of cases by health facilities

**Distribution by location:** most bites were recorded at Nkayi Base Hospital (44 cases), followed by Madingou Base Hospital (9 cases), then Kingoue Integrated Health Centers, Loudima post and Loudima station with 4 cases. The other centres only recorded 3, 2 or 1 cases (Figure 4).

### Clinical aspects

**Distribution of bites according to bite sites:** the bite site was undetermined in 83.95% (68/81) of cases, 14% (11/81) of bites were on the lower limbs and 1% (1/81) involved the upper limbs and the head.

**Therapeutic aspects:** for treatment, victims received local care, analgesics, serum and tetanus vaccine (SAT and VAT) and antibiotic therapy. An anticoagulant (Lovenox) had been administered to a patient. The treatment administered to 30.38% of cases was not specified. Antivenom was lacking in all health facilities.

## Discussion

This study documents cases of snakebite recorded in the healthcare facilities of Bouenza over a period of thirteen years. Our goal was to gather data on snakebite incidence for a region where few data, and no recent data, currently exist.

The number of snakebites recorded in healthcare facilities in Bouenza during 2009-2021 (81) and the incidence of snakebite calculated from this number (18.62 per 100,000) are lower than expected. These estimates were much lower than those reported elsewhere in Africa [11] and lower than those reported in Congo, in 1986: an incidence of between 120 and 450 snakebites per 100,000 inhabitants per year in forests and 11.5 bites per 100,000 inhabitants in urban areas [4]. Possible reasons for this lower than expected incidence may include limitations of the study such as incomplete record-keeping in under-resourced clinics, or that snakebite victims in Bouenza are not seeking treatment in healthcare facilities. Age of victims in our study ranged from 4 to 70 years old, with adults (age 25-55) most affected. Our results are lower than those obtained by retrospective studies in Gabon [12], and in Guinea [11,13]. High incidence of snakebite in adults could be explained by the fact that adults are the main participants in field and livestock work which brings them into contact with snakes.

In our study, children (age 4-15) represent 11.11% of victims (9/81). A study from Morocco found that children under 15 represented 31.7% of cases [14]. A study from Burkina Faso [15] which included only pediatric patients (age 0-14) found that among children, the age group most affected was 10-14 with 56.8% of cases. This study showed that males and females were approximately equally affected with bites to males representing 50.63% of the cases recorded, and adults representing 54.32% of the cases. Several studies from West Africa have found adult men to be most affected by snakebite [16-21]. These studies found that adult men have activities (clearing, field preparation and weeding) that bring them into close contact with the habitat of snakes [21], which explains the higher incidence of bites. Our study was retrospective, the circumstances of the occurrence of snakebites were not always known.

In some years, women were more affected by snakebites. This phenomenon could be explained by the fact that in addition to fieldwork, women are bitten during wood collection and housework around chores [22].

The site of the bite is an important element in understanding the epidemiology of accidents due to snakebites [23]. In the present study, the lower limb is the site of 13.54% of the bites reported while 83.95% of cases did not specify the size of the bites. By taking the lower limbs as the main site of bite cases, we find that our results are lower than those obtained by many authors: [17] in Senegal who found 65%; [24], who found 77.27% in Ivory Coast; [15] in Burkina found 51.4%. Other authors have found even higher lower limb snakebite rates: 84% [25] in Ivory Coast), 83% [26] in Morocco; [18] in Mali and 90% [27] in Ivory Coast. This particular bite site is explained by the fact that the lower limbs are most exposed to contact with snakes and are often not very well protected. This is consistent with crawling which is the mode of locomotion of snakes at ground level. An exception we recorded was a case of a man being bitten on the head by a tree snake. Other authors have reported cases of bites on the hands or forearm with rates of 62.5% [28] and 80% [29]. These bites were associated with efforts to kill, handle or play with the snake (e.g. children). In our study, most bite cases (80.25%) were recorded during the rainy season. This result is consistent with reports from West Africa which found that more bites occur more during the rainy season [16,20,22,30] than the dry season. These bites mainly involve adults. The agricultural activities carried out during the rainy season could explain this preponderance of cases among adults. In addition, snakes are more active during the rainy season (mating, hunting), which favours human-snake encounters. In the departments where our study was conducted, there was no protocol for the management of bites and envenomations due to snakebites. However, the treatment of snakebites in health facilities in Bouenza was based on symptomatic treatment. It consisted of locally treating the site of the bite. In some cases, painkillers and antibiotics and sometimes anti-tetanus drugs were administered to people who had been bitten by the snakes. A study from Benin [31], reported cases of symptomatic treatment similar to those practised in Bouenza and which consists of the administration of analgesics, tetanus shots and antibiotics. In addition, some bite victims received infusions and blood transfusions. In the services surveyed during this study, no case of administration of anti-venom serum (SAV) to patients who had been victims of snakebites was recorded. This is due to the fact that anti-venom serum is expensive and rare in most healthcare facilities in Congo and particularly in those of Bouenza. In our study, half of the victims had received antibiotic treatment for prophylactic or curative purposes. Similar practices were noted in the study by [31]. The use of antibiotics is justified by the fact that the environment where the bite takes place is generally soiled, also by scarification practices often carried out under septic conditions, but also by the presence of bacteria responsible for superinfection in the oral cavity of snakes [32]. This also explains the serotherapy and/or tetanus vaccination in 30.38% of the cases recorded during our study. Tetanus prophylaxis will be practiced according to the usual rules for any bite caused by an animal [33].

The fatality rate in this study was 9.9% (8/81 victims). This is higher than an earlier study from Congo [4] which found that the fatality rate ranged from 1 to 6.6% The rate observed in this study is consistent with those recorded in most sub-Saharan African countries. However, this rate can vary from 2 to 18% depending on the country, its population density and its snake fauna [34]. Snakebites are common in the Congo, as they are in most tropical countries, and victims may consult the healthcare centre only in case of complications resulting in a higher fatality rate in healthcare centre records [35].

**Limitations:** the study had its limitations: problems with recording data and archiving records in all healthcare facilities, registers often poorly completed or even torn. The circumstances of the bite were not recorded. The type of snake was not recorded although the green mamba is much-implicated in Loudima. The sample was small and could not retrospectively obtain true incidence rates. The data set used was not designed for research and therefore made it difficult for investigators to get some useful information such as clinical parameters: vital signs, limb conditions including examination findings etc. which are the basis for the choice of management in snakebites. The retrospective nature of our study constitutes a limitation. The low incidence rate observed in this study between 2009 and 2021 may reflect limitations of the record-keeping from which the data were drawn, or that many snakebite victims are not seeking care at healthcare centres.

## Conclusion

Our study gives an idea of the distribution of snakebites in the department of Bouenza in the Republic of Congo. Most bite cases are concentrated in Nkayi Sub-Prefecture. The recorded snakebites occurred in adults during the most favourable season for agricultural activities. Thus, the working adult population is particularly impacted. This makes snakebite cases a public health concern. All the cases for which the site of the bite is known are to the lower limbs. In most cases, the site of the bite remains undetermined. The care of the victims is essentially symptomatic due to the lack of anti-venom serums in all the healthcare facilities of the Department of Bouenza. Management consisted of the administration of steroidal or non-steroidal anti-inflammatory drugs, antibiotics, analgesics and anti-tetanus serum. A prospective evaluation associated with epidemiological surveys within populations is necessary to update epidemiological information. This information will be used for decision-making on training and capacity-building measures for healthcare personnel in the management of cases of snakebites and envenoming.

### What is known about this topic

- *Snakebites have been recognized as a neglected tropical disease by the WHO; each year, 5.4 million snake bites occur resulting in 1.8 to 2.7 million cases of envenoming;*
- *In many countries where snakebites are common, health systems lack the infrastructure and resources to collect solid statistical data on this problem*.

### What this study adds

- *This study provides new data from a region where few data, and no recent data, exist, providing a first step toward deeper investigations on the impact of snake bites with the eventual goal of the establishment of a center of anti-venom serums;*
- *It highlights the difficulties encountered by therapists in Congo in the management of snakebite cases due to the lack of availability of anti-venom serums*.
